# The prevalence and correlates of biomarker positive unhealthy alcohol use among women living with and without HIV in San Francisco, California

**DOI:** 10.1371/journal.pone.0308867

**Published:** 2024-10-04

**Authors:** Jennifer P. Jain, Yifei Ma, Carol Dawson-Rose, Glenn-Milo Santos, Alvina Han, Jennifer Price, Judith A. Hahn, Phyllis C. Tien

**Affiliations:** 1 Department of Community Health Systems, University of California, San Francisco, San Francisco, California, United States of America; 2 Department of Medicine, University of California, San Francisco, San Francisco, California, United States of America; 3 Department of Epidemiology and Biostatistics, University of California, San Francisco, San Francisco, California, United States of America; 4 Department of Veteran Affairs Medical Center, Medical Service, San Francisco, CA, United States of America; Emory University, UNITED STATES OF AMERICA

## Abstract

The objective of this study was to identify the prevalence and correlates of phosphatidylethanol (PEth) levels suggestive of unhealthy alcohol use among women living with and without HIV who self-reported no or low-risk drinking. We analyzed data from a cross-sectional study among women enrolled in the San Francisco Bay Area site of the Women’s Interagency HIV Study (WIHS). Between October 2017 and March 2018, PEth was tested from dried blood spots in 192 women enrolled in the San Francisco site of the WIHS. Using multivariable logistic regression, we identified the correlates of PEth levels suggestive of unhealthy alcohol use (>50 ng/ml) among the 168 women who reported no or low-risk drinking (<7 drinks per week) in the past six months, while controlling for age in years and race/ethnicity. Among the 168 women in the analysis sample, the median age was 55; 51% identified as Black/African American, 47% were living with HIV and 28% had PEth levels ≥50 ng/ml which are suggestive of unhealthy alcohol use. Factors independently associated with PEth levels ≥50 ng/ml in adjusted models were: identifying as Black/African American (adjusted odds ratio [aOR] = 8.34, 95% CI = 2.06–33.72), having an alanine transaminase to aspartate aminotransferase ratio > 1 (aOR = 3.10, 95% CI = 1.18–8.13), higher high-density lipoprotein levels (aOR = 1.31 per 10 mg/dL increase, 95% CI = 1.01–1.70), and consuming a greater number of drinks per week in the past six months (aOR = 1.40, 95% CI = 1.10–1.78). Nearly a third of women in this study had PEth levels suggestive of unhealthy alcohol use and potentially under-reported their use. To optimize alcohol related health care, there is a need to consider approaches to improve ascertainment of unhealthy alcohol use, especially among Black/African American women and those living with liver disease, so that interventions can be initiated.

## Introduction

Alcohol use disorder is the most prevalent type of substance use disorder worldwide, and is associated with increased morbidity and mortality, particularly among people living with HIV (PLWH) [1, 2]. Between 2011 and 2015, unhealthy alcohol use was responsible for 95,000 deaths and 2.8 million years of potential life lost in the United States (US) alone [3, 4]. In the Women’s Interagency HIV Study (WIHS), the largest study examining the epidemiology of HIV among women in the US, nearly half of the study participants reported using any alcohol and almost a quarter reported unhealthy alcohol use, also referred to as heavy or hazardous drinking, defined as drinking more than seven drinks per week in the past six months [5, 6].

Alcohol use research often relies upon self-reported measures of alcohol consumption which are subject to both recall bias and social desirability bias (usually under-reporting) and may lead to inaccurate estimates of alcohol consumption [7–10]. To improve the accuracy of alcohol use measurement, there is a need to leverage biomarkers of alcohol consumption such as phosphatidylethanol (PEth) in conjunction with self-reported measures of alcohol use. Using biomarkers like PEth among hidden populations such as women living with HIV (WLHIV) and demographically similar women without HIV who use alcohol in the US, is especially important because less is known about the correlates of high PEth levels in this population [5, 11–13].

PEth is a direct alcohol metabolite that has shown promise in detecting under-reported alcohol use among PLWH [8, 14, 15]. PEth is a phospholipid that is formed only in the presence of alcohol and is detected in the membrane of red blood cells [16]. PEth levels demonstrate high sensitivity (88%) and specificity (89%) in detecting any alcohol consumption within 21 days prior to testing among PLWH [15]. However, PEth levels in blood can be influenced by the amount and frequency of alcohol consumption, therefore recent alcohol use can contribute to higher PEth levels [17]. Also, it is important to note that the agreement between PEth levels in blood and self-reported alcohol use varies across different populations and study designs [8, 14, 18, 19]. Furthermore, less is known about the correlates of biomarker positive unhealthy alcohol use among women living with HIV and demographically similar women without HIV who report no or low-risk drinking in the US.

To reduce these gaps in research, we studied women living HIV and women with similar risk profiles living without HIV in San Francisco, California to identify the factors associated with having a PEth result suggestive of unhealthy alcohol use (>50 ng/mL) [20, 21]. Further, in order to identify those who potentially under-reported their alcohol use, we restricted the analysis sample to women who reported no or low-risk drinking in the past six months. We hypothesized that the strongest risk factors for having a PEth result suggestive of unhealthy alcohol use (>50 ng/mL) and potentially under-reported alcohol use, would be a history of viral hepatitis and metabolic-associated liver disease, which are common among PLWH. This hypothesis is based on our understanding that individuals living with liver disease or injury are more likely to receive alcohol use reduction messages from healthcare providers that may impact their self-report [22, 23].

## Methods

### Study population

The WIHS (now integrated with the Multicenter AIDS Cohort Study (MACS) to become the MACS-WIHS Combined Cohort Study [MWCCS]) was established in 1994 to investigate the course of HIV and associated conditions among women from ten study sites in the US: the Bronx and Brooklyn, NY; Chicago, IL; San Francisco, CA; Los Angeles, CA; Washington D.C.; Atlanta, GA; Chapel Hill, NC; Miami, FL; Jackson, MS; and Birmingham, AL. WIHS is the oldest and largest prospective cohort study of WLHIV in the world and the leading study examining the epidemiology of HIV among women in the US [20]. Details on recruitment, retention and profile characteristics of women in the WIHS have been published previously [20].

### Study procedures

WIHS procedures and protocols are described fully elsewhere [24]. Briefly, at each semiannual WIHS visit, participants completed an interviewer-administered questionnaire collecting information on sociodemographic factors, health history, medication use, alcohol use, and cigarette/tobacco smoking. Participants also completed a physical exam, phlebotomy for testing of CD4 count, metabolic parameters, and in WLHIV, HIV RNA level. At the WIHS enrollment visit, all women were screened for HIV and HCV antibodies. In those who were HIV seronegative, HIV antibodies were tested annually. Between October 1, 2017 and March 31, 2018, women from the San Francisco Bay Area WIHS site were asked to participate in a cross-sectional ancillary study to measure PEth at the time of their semiannual WIHS research visit.

### PEth assay

Dried blood spot (DBS) samples prepared from blood collected at the time of the WIHS visit were sent to the United States Drug Testing Laboratories in Des Plaines, IL for PEth quantification of the PEth 16:0/18:1 analog using a previously reported method, with limit of quantification of 8 ng/ml [25]. PEth detects alcohol exposure for up to 21 days prior to the date of sample collection [26].

### Ethical considerations

All participants provided written informed consent and study procedures took place in a private setting. For the present study, all study procedures and protocols were reviewed and approved by the Institutional Review Board at the University of California, San Francisco.

### Measures

#### Self-reported measures of alcohol use

Participants were asked how often they drank alcohol in the past six months. From this the following drinking categories were created; light drinking (yes/no) was defined as reporting more than 0 but less than 3 drinks per week, moderate drinking (yes/no) was defined as 3–7 drinks per week and unhealthy drinking (yes/no) was defined as consuming more than 7 drinks per week [27]. We also measured binge drinking (yes/no) which was defined as drinking 4 or more drinks on one occasion in the prior six months [28].

#### Outcome measure and PEth levels

We measured PEth levels for all participants and created a binary measure of PEth levels ≥50 ng/ml (yes/no) which served as our primary outcome of interest. We used this threshold as it has been used previously as a cutoff point for unhealthy alcohol use with high sensitivity and specificity [10, 17, 29, 30].

#### Covariates

Covariates included sociodemographic factors [age, race/ethnicity (White, Black/African American, Hispanic, and Other), level of education (less than high school, high school, more than high school), annual income (<$12,000, $12,000-$30,000 and >$30,000); HIV and HCV serostatus, and in those living with HIV, CD4 cell count (cells/mm^3^) and HIV viral load (VL); markers of liver injury (continuous ALT and AST values, an ALT:AST ratio >1, a marker of fatty liver disease [31], and indirect serum markers of liver fibrosis including the aspartate aminotransferase to platelet ratio index (APRI) and Fibrosis-4 (FIB-4) scores [32, 33]; cigarette/tobacco smoking history (current, former, never); and metabolic parameters (BMI in kg/m^2^, waist circumference in centimeters, lipid parameters (low density lipoprotein (LDL), high-density lipoprotein (HDL), triglycerides) in mg/dL and diabetes (yes/no) defined as per WIHS standardized criteria [34].

### Statistical analysis

To identify those at risk of biomarker positive unhealthy alcohol use, we restricted the analysis sample to the 168 women who reported no or low-risk drinking (<7 drinks/week) in the past six months. We estimated the proportion with PEth ≥50 ng/ml and calculated 95% confidence intervals. We then compared sociodemographic, behavioral, and clinical characteristics in women with PEth levels >50 ng/ml versus PEth levels <50 ng/ml. We used Chi-square tests for categorical and dichotomous variables and pooled variance T-tests or Mann-Whitney U tests for normally and non-normally distributed continuous variables, respectively (Table 1). To assess whether PEth levels increased with the self-reported number of drinks consumed per week in the past six months, a Spearman correlation coefficient was calculated.

**Fig 1:**
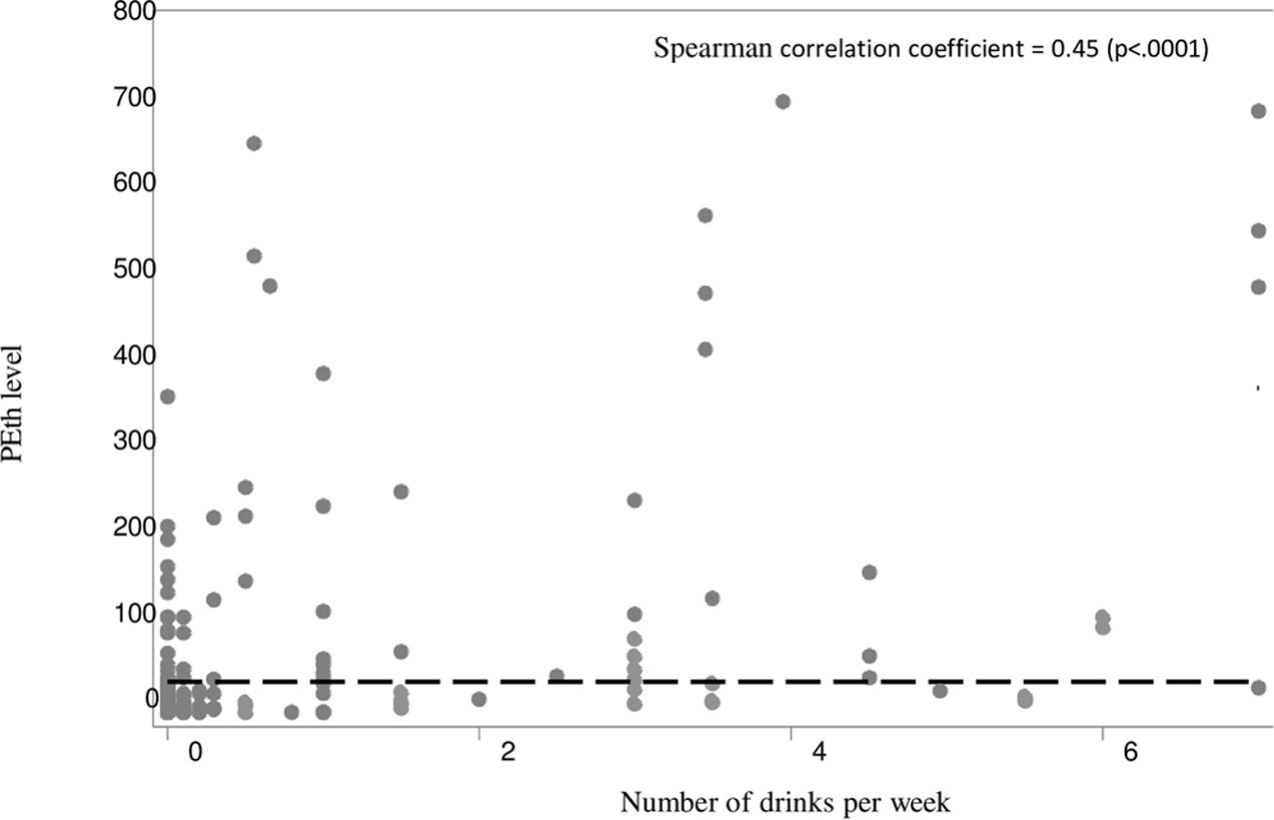
PEth levels (16:0/18:1, ng/mL) by the self-reported number of drinks consumed per week in the past six months among women in the Women’s interagency HIV study in San Francisco, CA (N = 192).

**Table 1:** Baseline sociodemographic, behavioral, and clinical characteristics by PEth levels <50 ng/mL vs. ≥50 ng/mL, (N = 168).

Variable	Overall	PEth	PEth	*P-value*
	(N = 168)	<50 ng/mL	>50 ng/mL	
		(n = 120)	(n = 48)	
		n (%)	n (%)	
	n (%) or	n (%) or Median (IQR)	n (%) or Median (IQR)	
	Median (IQR)			
***PEth level (ng/ml)***	14 (0.0–65)	5.5 (0.0–17.5)	154 (94.5–381)	<0.001^1^
***Demographics***				
Age (years)	55.7 (48.6–61.8)	56.9 (48.4–61.9)	55.9 (48.6–61.8)	0.71^1^
*Race*				
White	36 (21.4%)	29 (24.2%)	7 (14.6%)	
Black or African American	86 (51.2%)	54 (45.0%)	32 (66.7%)	
Hispanic	25 (14.9%)	20 (16.7%)	5 (10.4%)	
Other	21 (12.5%)	17 (14.2%)	4 (8.3%)	0.092²
*Education*				
Less than high school	44 (26.2%)	32 (26.7%)	12 (25.0%)	0.11²
High school	61 (36.3%)	38 (31.7%)	23 (47.9%)	
More than high school	63 (37.5%)	50 (41.7%)	13 (27.1%)	
*Income*				
<$12,000	72 (42.9%)	49 (40.8%)	23 (47.9%)	0.64²
$12,000-$30,000	50 (29.8%)	36 (30.0%)	14 (29.2%)	
>$30,000	46 (27.4%)	35 (29.2%)	11 (22.9%)	
***Cigarette use***				
Current cigarette use	64 (38.1%)	45 (37.5%)	19 (39.6%)	0.39²
Past cigarette use	47 (28.0%)	37 (30.8%)	10 (20.8%)	
Never smoked cigarette	57 (33.9%)	38 (31.7%)	19 (39.6%)	
***Alcohol use past six months***				
Abstained	76 (45.2%)	64 (53.3%)	12 (25.0%)	< .0001²
Light drinking (>0, <3 drinks/wk)	61 (36.3%)	43 (35.8%)	18 (37.5%)	
Moderate drinking (3–7 drinks/wk)	31 (18.5%)	13 (10.8%)	18 (37.5%)	
Binge drinking (>4 drinks on one occasion)	29 (17.3%)	12 (10.0%)	17 (35.4%)	< .0001²
***Metabolic***				
BMI (kg/m^2^)	29.2 (25–37.4)	29.4 (25.1–36.6)	28.9 (24.7–38.5)	0.67°
Waist circumference (cm)	102 (90.4–116)	102 (90.7–117)	104 (90–115)	0.72°
Diabetes	35 (20.8%)	22 (18.3%)	13 (27.1%)	0.21°
LDL (mg/dL)	97 (73–119)	96.5 (73–116)	97 (73–126)	0.67°
HDL (mg/dL)	58 (47–71)	57 (45.5–69.5)	64 (50–74)	0.039°
Triglycerides (mg/dL)	98 (71–139)	99.5 (71–140)	95 (72–139)	0.99°
***HIV and HCV status***				
HIV+/HCV ab+	36 (21.4%)	25 (20.8%)	11 (22.9%)	0.61²
HIV+/HCV ab-	80 (47.6%)	55 (45.8%)	25 (52.1%)	
HIV-/HCV ab+	14 (8.3%)	12 (10.0%)	2 (4.2%)	
HIV-/HCV ab-	38 (22.6%)	28 (23.3%)	10 (20.8%)	
HIV Viral load (copies/ml) (IQR)^3^	34 (20–108)	33 (20–110)	35 (20–108)	0.38°
CD4 (cells/mm^3^) (IQR)^3^	742 (464–898)	771 (489–919)	619 (413–843)	0.96°
***Liver function***				
Viral hepatitis (HCV or HBV)	12 (7.1%)	9 (7.5%)	3 (6.3%)	0.78²
AST	19.5 (16–24)	19 (16–23.5)	20.5 (16–24.5)	0.047°
ALT	17 (13–23)	16 (12.5–22)	18 (13.5–24.5)	0.14°
ALT >38 U/L	7 (4.2%)	4 (3.3%)	3 (6.3%)	0.39²
ALT:AST >1	45 (26.8%)	27 (22.5%)	18 (37.5%)	0.047^2^
APRI	0.23 (0.175–0.31)	0.22 (0.17–0.31)	0.24 (0.18–0.34)	0.067°
FIB4	1.03 (0.775–1.44)	1.03 (0.77–1.43)	1.04 (0.79–1.59)	0.18°

Notes:

^0^Based on pooled variances t-tests

^1^Based on Mann-Whitney tests / Wilcoxon Rank-Sum tests

^2^Based on Chi-square tests

^3^For participants living with HIV only

IQR = interquartile range

Some percentages may reflect denominators smaller than the total N, this is due to missing data

To explore the correlates of PEth levels ≥50 ng/ml, we first used bivariable logistic regression to model unadjusted associations of demographic factors, HIV and HCV status, markers of liver disease, metabolic parameters, and alcohol use with PEth levels ≥50 ng/ml. Multivariable logistic regression models were then built by mutually adjusting for each variable that had a p-value of <0.10 in bivariable models and adjusting for age in years and race/ethnicity (White [reference group], Black/African American, Hispanic, and Other) (Table 2). We explored these variables to identify the demographic predictors of women with PEth levels ≥50 ng/ml who reported no or low-risk drinking, which we consider biomarker positive unhealthy alcohol use. In addition, we sought to test our primary hypothesis which was that; among women who report no or low alcohol use, those who have a liver-affecting disease would having a higher odds of having PEth levels ≥50 ng/ml or biomarker positive unhealthy alcohol use, suggesting under-reporting, compared to those with no such diagnosis.

**Table 2:** Bivariate and multivariable logistic regression models examining factors associated with PEth levels ≥50 ng/ml among women reporting no or low-risk drinking in the Women’s interagency HIV study in San Francisco, CA (N = 168).

Variable	Unadjusted Odds Ratio	Adjusted Odds Ratio
	(95% CI)	(95% CI)
Age	1.00 (0.97–1.04)	1.01 (0.97–1.06)
***Race/Ethnicity***		
White	Reference group	Reference group
Black/African American	2.45 (0.96–6.24)	8.34 (2.06–33.72)**
Hispanic	1.03 (0.28–3.73)	2.85 (0.52–15.56)
Other^‡^	0.97 (0.24–3.82)	4.50 (0.75–27.09)
*HIV and HCV status*		
HIV+/HCVab+	1.23 (0.44–3.39)	2.54 (0.69–9.61)
HIV+/HCV-	1.27 (0.53–3.01)	1.80 (0.62–5.20)
HIV-/HCVab+	0.46 (0.08–2.45)	0.23 (0.02–2.68)
***Hepatic function***		
ALT:AST>1	2.06 (1.001–4.26)*	3.10 (1.18–8.13)*
***Metabolic function***		
HDL per 10 (mg/dL)	1.23 (1.001–1.04)*	1.31 (1.01–1.70)*
***Self-reported Alcohol use***		
Number of drinks per week	1.44 (1.18–1.74)***	1.40 (1.10–1.78)**

Notes:

^‡^Other includes, Asian, Pacific Islander, Native American, Alaskan, and other study participants

Variables with a p-value that was <0.10 in bivariate models for were candidates for inclusion in the multivariable models

Multivariable models controlled for age and race/ethnicity

Levels of significance are denoted with an asterisk as follows; <0.05*, <0.01** and <0.001***

These analyses were performed on complete cases only, so all participants with missing outcome or covariate data were excluded from the analyses. Overall, there was a minimal amount of missing data (<5%), and individuals who were excluded due to missing data did not differ on key variables of interest. All analyses were performed in SAS 9.4 (SAS Institute Inc., Cary, NC).

## Results

A total of 238 women were seen at the San Francisco Bay Area WIHS site for their semi-annual research visit that took place between October 1, 2017 and March 31, 2018. Among these, 192 had DBS collected for PEth testing, and among these 168 reported no or low-risk drinking in the past six months and were included in this study. Study characteristics between the 46 women who did not have DBS collected for PEth testing and the 192 who were considered for analysis did not differ significantly (S1 Table).

Among the 168 women included in this study, the median age was 55 (IQR = 48, 61) and over half (51%) identified as Black or African American. Approximately a third (36%) reported completing high school and 42% reported earning less than $12,000 annually. Nearly half (47%) were living with HIV and 40% were current cigarette smokers. Overall, 45% reported abstaining from alcohol, 36% reported light drinking, 18% reported moderate drinking, and 17% reported binge drinking in the past six months. The median PEth level in this sample was 14 ng/mL (IQR: <Limit of quantification [LOQ]-65) and in general PEth appeared to increase with the self-reported number of drinks consumed per week in the past six months (Spearman correlation coefficient 0.45, p<0.001) (Fig 1). A total of 48 women out of 168 (28%) had PEth levels ≥50 ng/ml, which is suggestive of unhealthy alcohol use and potential under-report given they reported no or low-risk drinking.

Women with PEth levels ≥50 ng/ml were more likely to engage in moderate drinking, binge drinking, had higher HDL levels and higher AST levels compared to women with PEth levels <50 ng/ml. In multivariable models, factors significantly associated with PEth levels ≥50 ng/ml were: identifying as Black or African American (adjusted odds ratio [aOR] = 8.34, 95% CI = 2.06–33.72, *p* = 0.003), having an ALT:AST ratio greater than 1 (aOR = 3.10, 95% CI = 1.18–8.13, *p* = 0.021), higher HDL (aOR = 1.31 per 10 mg/dL increase, 95% CI = 1.01–1.70, *p* = 0.036), and reporting a greater number of drinks consumed per week in the past six months (aOR = 1.40, 95% CI = 1.10–1.78, *p* = 0.005).

## Discussion

This study among women living with and without HIV enrolled in the San Francisco Bay Are WIHS site, found that nearly one third of women reporting no or low-risk drinking had PEth levels ≥50 ng/ml, which are suggestive of unhealthy alcohol use and potentially under-reported alcohol consumption. In addition, this study identified the following correlates of PEth levels ≥50 ng/ml; identifying as Black/African American, having an ALT:AST ratio>1, having higher HDL levels, and consuming a greater number of drinks per week in the past six months. In this study, HIV was not associated with having PEth levels ≥50 ng/ml. These findings may have important implications for clinical interventions that address alcohol use and comorbid conditions among US women living with HIV and demographically similar women living without HIV.

Consistent with our main hypothesis, we found that women with an ALT:AST>1, which is indicative of fatty liver disease, had a higher odds of having PEth levels ≥50 ng/ml. This finding is consistent with literature noting that alcohol use may be under-reported among those with liver disease or liver injury, because individuals with these conditions are often counseled to reduce their alcohol use, and therefore may feel pressure to report lower levels of alcohol use (i.e., socially desirability bias) [22, 23]. It is also important to note that while liver disease does impact alcohol metabolism it does not impact PEth levels in blood. Based on this, we recommend using PEth to measure alcohol use among individuals with liver disease/injury, and referring those with PEth ≥50 ng/mL to alcohol treatment. Our finding adds to the literature [35, 36] demonstrating the relationship between liver injury, alcohol consumption and self-report and PEth.

Our finding that Black/African American women (versus White women) had a higher odds of having PEth levels ≥50 ng/ml, is an incidental finding because race/ethnicity was controlled for in the multivariable analyses. This finding should be interpreted within the context of systems of power and privilege (e.g. racism and sexism) that fuel disparities in poor health outcomes among Black/African American women who engage in substance use [37–39]. According to prior research, the burden of adverse health outcomes due to alcohol use disorder is greater among Black/African American women compared to White women, and is partially driven by experiences of racial discrimination and alcohol use stigma [40]. Thus, it is possible that among the Black/African American women in our study, anticipated alcohol use stigma and anticipated racial discrimination led them to under-report their alcohol use as a mechanism to avoid these negative experiences (i.e. socially desirability bias).

Future alcohol use research among Black/African American women, should consider using strategies to reduce social desirability bias including, establishing rapport using techniques rooted in harm reduction such as non-judgement and acceptance, and addressing underlying motivations for socially desirable reporting [41, 42]. Moreover, from a broader perspective, promoting social justice among women who fill this unique intersectional space (Black/African-American women who engage in alcohol use) is also critical. Finally, our research highlights the need for culturally sensitive and gender specific interventions to reduce alcohol use and improve engagement in healthcare among Black/African American women in the US [43–48].

The moderate correlation between the self-reported number of drinks consumed per week and PEth levels, may reflect some biological heterogeneity in alcohol metabolism that can affect PEth levels [49]. Alternatively, this finding may be further evidence of under-reported unhealthy alcohol use, which may be either by minimizing one’s report of drinking or outright denying their drinking. In this case, we emphasize again the need to utilize counseling methods to reduce social desirability bias including rapport building, creating a non-judgmental space for data collection and clearly defining the role of a study participant [41, 42].

We also found that women with higher HDL cholesterol levels had a higher odds of PEth levels ≥50 ng/ml. This finding was anticipated because prior studies have suggested that light and moderate drinking can increase HDL levels through an increase in the transport rate of apolipoproteins [50, 51]. Our research confirms that a similar relationship between HDL cholesterol levels and PEth levels exists among women living with HIV and women with similar risk profiles living without HIV in the US.

### Limitations

This study should be interpreted in the context of certain limitations. This was a cross-sectional study therefore we cannot identify temporal trends or determine the causal impact of the measured exposures on our outcome of interest. It is possible that residual confounding related to unmeasured factors affected our results. Many of our estimates have wide confidence intervals, reflecting the modest sample size, and should be interpreted with caution. We did not measure social desirability bias or alcohol use stigma which limits our understanding of the role these factors play. Future studies should consider measuring such constructs as these may shed more light on what leads to unhealthy alcohol use and under-reported alcohol use. Recent binge alcohol use may have contributed to some of the elevated PEth levels. Alcohol use surveys were administered every six months, therefore self-reported data on alcohol consumption were only collected semi-annually. However, PEth detects alcohol consumption over the past 21 days, which limits our ability to provide an accurate correlation between self-reported alcohol use and PEth levels. Finally, this study was conducted among women enrolled at a single WIHS site, however future investigations are planned among the larger MWCCS multisite cohort that will assess alcohol use over the past 7–30 days to establish a more accurate link between self-report and PEth levels in blood.

## Conclusions

Despite these limitations, our study shows that PEth may improve the detection of unhealthy alcohol use and potential under-report among US women living with and without HIV. Findings from this study underscore the need to leverage biomarkers of alcohol consumption to improve alcohol use measurement among hidden populations like women living with HIV and women without HIV with similar risk profiles. Further, this study highlights the need for female-centered interventions that leverage non-judgmental counseling methods rooted in harm reduction and motivational interviewing techniques to meet women where they are and empower them to engage in the change process [52].

## Supporting information

S1 Table Study characteristics among women with no DBS samples collected who were not included in the study compared to those with DBS samples collected who were considered for the analysis. (DOCX)
