# Supplementary material for: The prevalence and correlates of biomarker positive unhealthy alcohol use among women living with and without HIV in San Francisco, California
Source: PLoS One. 2024 Oct 4;19(10):e0308867. doi: 10.1371/journal.pone.0308867 (PMC11451982; doi:10.1371/journal.pone.0308867)
Supplement: S1 Table — (DOCX) [file pone.0308867.s001.docx]

| **Study characteristics among women with no DBS samples collected who were not included in the study compared to those with DBS samples collected who were considered for the analysis** | | | | |
| --- | --- | --- | --- | --- |
|  | **Overall (N=238)** | **Not included in PEth study (N=46)** | **Included in PEth Study (N=192)** | *P-value* |
| ***Age*** |  |  |  |  |
| Mean (SD) | 53.1 (9.51) | 52.3 (11.0) | 53.3 (9.14) | 0.587 |
| Median [Min, Max] | 53.0 [35.0, 83.0] | 51.0 [35.0, 78.0] | 53.0 [36.0, 83.0] |  |
| Median (IQR) | 53.0 (13.8) | 51.0 (16.8) | 53.0 (12.0) |  |
| ***Race*** |  |  |  |  |
| White | 45 (18.9%) | 7 (15.2%) | 38 (19.8%) | 0.153 |
| Black or African American | 133 (55.9%) | 32 (69.6%) | 101 (52.6%) |  |
| Hispanic | 32 (13.4%) | 5 (10.9%) | 27 (14.1%) |  |
| Other | 28 (11.8%) | 2 (4.3%) | 26 (13.5%) |  |
| ***Education*** |  |  |  |  |
| Less than high school | 67 (28.2%) | 14 (30.4%) | 53 (27.6%) | 0.0745 |
| High school | 92 (38.7%) | 23 (50.0%) | 69 (35.9%) |  |
| More than high school | 79 (33.2%) | 9 (19.6%) | 70 (36.5%) |  |
| ***Cigarette use*** |  |  |  |  |
| Never smoker | 58 (24.4%) | 10 (21.7%) | 48 (25.0%) | 0.858 |
| Current smoker | 96 (40.3%) | 20 (43.5%) | 76 (39.6%) |  |
| Former smoker | 84 (35.3%) | 16 (34.8%) | 68 (35.4%) |  |
| ***Alcohol use*** |  |  |  |  |
| Abstainer | 96 (40.3%) | 20 (43.5%) | 76 (39.6%) | 0.949 |
| >0-7 drinks/wk | 112 (47.1%) | 20 (43.5%) | 92 (47.9%) |  |
| >7-12 drinks/wk | 6 (2.5%) | 1 (2.2%) | 5 (2.6%) |  |
| >12 drinks/wk | 24 (10.1%) | 5 (10.9%) | 19 (9.9%) |  |
| ***BMI*** |  |  |  |  |
| Mean (SD) | 27.6 (12.1) | 26.2 (12.8) | 27.9 (11.9) | 0.405 |
| Median [Min, Max] | 28.0 [0, 67.6] | 28.5 [0, 58.0] | 28.0 [0, 67.6] |  |
| Median (IQR) | 28.0 (12.0) | 28.5 (11.9) | 28.0 (11.9) |  |
| ***Diabetes*** |  |  |  |  |
| No diabetes | 190 (79.8%) | 36 (78.3%) | 154 (80.2%) | 0.927 |
| Diabetes | 48 (20.2%) | 10 (21.7%) | 38 (19.8%) |  |
| ***HIV status*** |  |  |  |  |
| Negative | 78 (32.8%) | 15 (32.6%) | 63 (32.8%) | 0.826 |
| Prevalent | 157 (66.0%) | 30 (65.2%) | 127 (66.1%) |  |
| Converter | 3 (1.3%) | 1 (2.2%) | 2 (1.0%) |  |
| ***APRI*** |  |  |  |  |
| Mean (SD) | 0.230 (0.424) | 0.224 (0.465) | 0.231 (0.415) | 0.928 |
| Median [Min, Max] | 0.170 [0, 4.46] | 0.125 [0, 2.84] | 0.180 [0, 4.46] |  |
| Median (IQR) | 0.170 (0.260) | 0.125 (0.233) | 0.180 (0.270) |  |
| ***FIB4*** |  |  |  |  |
| Mean (SD) | 0.993 (1.45) | 0.854 (1.17) | 1.03 (1.51) | 0.4 |
| Median [Min, Max] | 0.800 [0, 14.7] | 0.750 [0, 5.25] | 0.820 [0, 14.7] |  |
| Median (IQR) | 0.800 (1.26) | 0.750 (1.19) | 0.820 (1.27) |  |
